# The Impact of Compact Layer in Biphasic Scaffold on Osteochondral Tissue Engineering

**DOI:** 10.1371/journal.pone.0054838

**Published:** 2013-01-28

**Authors:** Hu Da, Shuai-Jun Jia, Guo-Lin Meng, Jian-Hua Cheng, Wei Zhou, Zhuo Xiong, Yun-Jing Mu, Jian Liu

**Affiliations:** 1 Institute of Orthopaedics and Traumatology, Xijing Hospital, The Fourth Military Medical University, Xi’an, China; 2 The 82nd hospital of PLA, Huaian, China; 3 Shannxi Hospital of Armed Police Force, Xi’an, China; 4 Department of Mechanical Engineering, Tsinghua University, Beijing, China; 5 Zhan Tan Si Clinic of 309 Hospital of PLA, Beijing, China; University of California, San Diego, United States of America

## Abstract

The structure of an osteochondral biphasic scaffold is required to mimic native tissue, which owns a calcified layer associated with mechanical and separation function. The two phases of biphasic scaffold should possess efficient integration to provide chondrocytes and osteocytes with an independent living environment. In this study, a novel biphasic scaffold composed of a bony phase, chondral phase and compact layer was developed. The compact layer-free biphasic scaffold taken as control group was also fabricated. The purpose of current study was to evaluate the impact of the compact layer in the biphasic scaffold. Bony and chondral phases were seeded with autogeneic osteoblast- or chondrocyte-induced bone marrow stromal cells (BMSCs), respectively. The biphasic scaffolds-cells constructs were then implanted into osteochondral defects of rabbits’ knees, and the regenerated osteochondral tissue was evaluated at 3 and 6 months after surgery. Anti-tensile and anti-shear properties of the compact layer-containing biphasic scaffold were significantly higher than those of the compact layer-free biphasic scaffold *in vitro*. Furthermore, *in vivo* studies revealed superior macroscopic scores, glycosaminoglycan (GAG) and collagen content, micro tomograph imaging results, and histological properties of regenerated tissue in the compact layer-containing biphasic scaffold compared to the control group. These results indicated that the compact layer could significantly enhance the biomechanical properties of biphasic scaffold *in vitro* and regeneration of osteochondral tissue *in vivo*, and thus represented a promising approach to osteochondral tissue engineering.

## Introduction

Articular cartilage defects are common outcomes of trauma and joint disease, generally leading to severe pain and functional impairment. Very often, subchondral bone undergoes degeneration with chondral defect at the same time. The underlying subchondral defect site is critical as it supports and integrates with the overlying neocartilage [1]. Therefore, lesions of subchondral bone should be treated together with the chondral defect for optimum results. Since the cartilage has a limited capacity of regeneration, [2] and the neocartilage is difficult to integrate with the degenerated subchondral bone *in vivo* [3], current treatment strategies including abrasion arthroplasty, subchondral bone drilling, chondrocytes implantation and microfracture commonly result in poor outcomes [4], [5]. Tissue engineering is the most promising method that combines seed cells, scaffolds and growth factors for repair of articular osteochondral defects [6]. Specifically, recent advancements in biphasic osteochondral scaffolds have been successfully engineered for use in osteochondral grafts [7]–[9]. Mimicking the natural structure of joint tissues, it consists of a superficial cartilaginous layer (corresponding to articular cartilage) and an underlying calcified tissue (corresponding to subchondral bone) [10]. Previously, integration of graft with defect site was generally carried out using sutures and/or applying a tissue adhesive. Although these methods had some efficacy, they suffered from insufficient bonding strength [11]. The biphasic osteochondral constructs are designed to be press-fit into pre-drilled cavities in the defect sites, are better anchored than chondral-only grafts, and are less likely to be displaced by shearing forces within the joint [12]. With previous mosaicplasty technique, the chondral-only engineered tissues integrated with surrouding native tissues by cartilage-to-cartilage interfaces, known to have a limited capacity to integrate [13]. The two regions of an osteochondral composite can be formed into a single continuous scaffold [8], [14], [15]. When the chondral-only engineered tissues are substituted by the biphasic osteochondral composites, the interfaces of integration between the grafts and with surrouding native tissues will be changed from cartilage-to-cartilage interfaces to bone-to-bone interfaces, which have a higher ability to integrate [10]. While initial successes have been achieved, several factors still limit the widespread implementation of these techniques. In these studies, the integration between chondral and bony phases cannot withstand the shear forces acting in the joint, often resulting in mechanical failures [12], [16]–[19]. Furthermore, the regeneration capacity of the seed cells in the biphasic scaffold is very low *in vivo*, making these techniques insufficient to meet the demands of clinical applications [10].

An improved understanding of the microstructure and substance metabolism of articular osteochondral tissue is required to address these issues and propose potential solutions. Articular cartilage is a multi-layered composite structure composed of superficial, middle, deep and calcified layers [20], [21]. The calcified layer serves as a vital physical barrier between the cartilage and subchondral bone, providing articular cartilage with independent environment. Due to the calcified layer, the oxygen and nutrient supplied is very different between articular cartilage and subchondral bone [22]. Without this barrier, the newly-born osteochondral tissue will be impaired [22]–[26]. Therefore, calcified layer is a prerequisite for functional and integrative repair of osteochondral tissue [27].

It is generally acknowledged that the architecture of the scaffold, a key property of the scaffolds, is the primary determinant for the interaction between the implant and recipient site [1]. Thus, ideal scaffold-based tissue engineering techniques should closely mimic the native physiological structure and environment [28]. The current design and fabrication methods were engineered to produce a novel biphasic scaffold connected by a compact layer that more closely resembles the native tissue. The resultant scaffold was composed of biphasic bony and chondral phases separated by an associated compact layer.

In our study, situated between the chondral and bony phases, the compact layer functioned as both a connector and insulator. The compact layer was fabricated from PLGA/β-TCP, a material with previously demonstrated biocompatibility [29], [30]. The chondral phase was derived from bovine decellularized articular cartilage extracellular matrix (ECM). The oriented structure of the chondral phase was designed to mimic the deep layer of cartilage oriented vertical to the surface in native tissue. Oriented scaffolds were prepared using a modified temperature gradient-guided thermal-induced phase separation (TIPS) technique followed by freeze-drying. After cross-linking by treatment with genipin solution, the chondral phase mechanical strength was sufficient to meet the requirements of cartilage regeneration [31]. The bony phase, possessing a core-sheath structure, was composed of a PLGA/β-TCP skeleton wrapped with Type I collagen. The core-sheath structure composite scaffold was fabricated using a controllable sprayer via low-temperature deposition manufacturing (LDM), forming a 3-D structural bone bracket. Its materials and structure of the scaffold were fairly biomimetic, and the production of the scaffold is based on the bionic principle through simulating human bone actual architecture [32]. Notably, previous *in vitro* and *in vivo* experiments have demonstraetd that both the bony and chondral phases exhibit significant cellular affinity and biocompatibility [31], [32].

Biphasic scaffolds have several features that suggest these techniques can be successfully applied for treatment of osteochondral defects, including (i) the ability of the compact layer to mimic the native calcified layer to provide chondrogenic and osteogenic induced BMSCs with optimal *in vivo* culturing environments, (ii) the inhibition of adverse infiltration of the bony and chondral phases [23]–[26], and. (iii) the mechanical properties to withstand typical forces acting on the joint. In addition, the compact layer plays a vital role in enhancing integration between the chondral and bony phases.

The objective of the study was: (i) comparing the biomechanical properties of compact layer-containing and compact layer-free biphasic scaffolds *in vitro*. (ii) evaluating histological and immunohistochemical morphology, gross appearance, micro tomograph imaging, and biochemical properties of the neogenetic tissue. In this study, the biphasic scaffolds combined with chondrogenic and osteogenic induced BMSCs were implanated into osteochondral defects of rabbits’ knees for 3 or 6 months. It was hypothesized that the compact layer would enhance the biomechanical properties of the biphasic scaffold. Moreover, provided with two independent living environment by the compact layer, the proliferation and differentiation of newly-born chondrocytes and osteocytes would be promoted. Findings from these studies will enable improvement of biomimetic biphasic scaffold design and promote formation of osteochondral tissue *in vivo*.

## Materials and Methods

### Materials and Synthesis of Compact Layer

**Figure 1:**
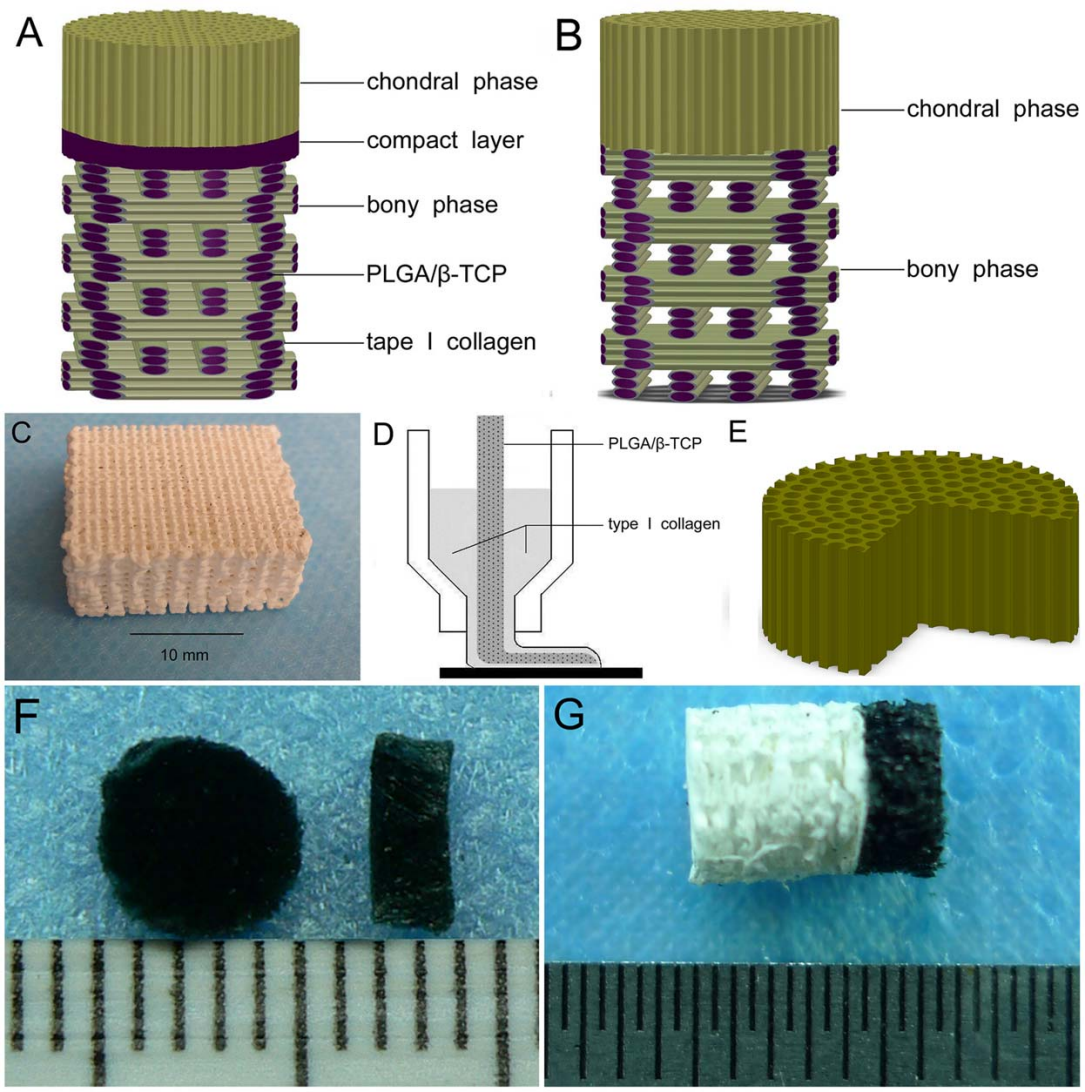
Schematic diagram and gross appearance. (A) schematic diagram of the compact layer-containing biphasic scaffold. (B) schematic diagram of the compact layer-free biphasic scaffold. (C) gross appearance of the bony phase. (D) the bony phase obtained by using a computer-controlled nozzle comprised of telescopic tubes. (E) schematic diagram of the chondral phase. (F) gross appearance of the chondral phase. (G) gross apperance of the bipasic scaffold.

The compact layer made from PLGA/β-TCP was fabricated by the phase separation process [33] in a low-temperature depostiton system (Designed by the Department of Mechanical Engineering of Tsinghua University). PLGA (LA/GA = 75/25, Mw = 104900, Mn = 92210, PI = 1.14) was purchased from Department of Medical Polymers Shandong Institute and β-TCP (particle size = 1.9±0.7 µm) was purchased from Chemical Material Factory of Wenzhou. Briefly, a 7∶3 PLGA to β-TCP mixture was dissolved in 1,4-dioxane at the room temperature resulting a homogeneous slurry of 25% PLGA/β-TCP. The slurry was extruded line by line onto the top of bony phase using a computer-controlled nozzle comprised of telescopic tubes in a low-temperature room. No space was presented between adjacent lines, which quickly solidified following extrusion. Frozen scaffolds were freeze-dried and 1,4-dioxane was sublimated, forming 0.5 mm thick compact layer (Fig. 1a).

### Preparation of Biphasic Scaffolds

Two types of 3-dimensional (3-D) porous biphasic scaffolds were constructed: compact layer-containing biphasic scaffolds (experimental group) and compact layer-free biphasic scaffolds (control group). Both groups were comprised of a chondral and bony phase. In the experimental group, the two phases were connected by the compact layer (Fig. 1A). Conversely, the two phases were connected directly in the control group (Fig. 1B). The bony phase was composed of a core-sheath structure, composite scaffold fabricated from a PLGA/TCP skeleton wrapped in type I collagen (Fig. 1A,B,C). This structure was obtained by rapid prototyping technique using a computer-controlled nozzle comprised of telescopic tubes, as previously described (Fig. 1D) [32]. The sponge-like chondral phase (Fig. 1E,F) was a, 3-D interconnected porous, oriented structure formed using bovine cartilage extracellular matrix-derived scaffold, constructed by a modified temperature gradient-guided thermal-induced phase separation (TIPS) technique, as previously described [31]. Independent fabrication processes were used for each of the two phases and the compact layer.

In the experimental group, the chondral phase and the bony phase were bonded by the compact layer through the dissolving-conglutination method. Briefly, the surface of the compact layer was dissolved by application of a 1,4-dioxane. The chondral phase was then placed onto the dissolved compact layer, and gentle pressure was evenly applied. Immediately, bonded biphasic scaffolds were frozen for 2 h at −80°C and subsequently lyophilized for 24 h. Finally, 1,4-dioxane was removed by sublimation, resulting in a firm bond between the two phases. The two phases were bonded directly using the same process in the control group.

Both biphasic scaffold types were processed into identical cylindrical structures (diameter = 5 mm). Chondral and bony phase heights were 2 mm and 4 mm, respectively (Fig. 1G). All biphasic scaffolds were sterilized by exposure to 20 kGy ^60^Co radiation before BMSCs implantation.

### Characterization of Compact Layer and Biphasic Scaffolds

Morphological structure of the scaffolds was observed by scanning electron microscopy (SEM; S-3400N, Hitachi, Tokyo, Japan). Micropores and microtubules diameters of the compact layer and biphasic scaffolds were determined according to the SEM image by averaging 50 of them [34].

The compact layer was fabricated alone into a 1×1×1 cm^3^ structure. The shape and size of biphasic scaffolds were described above. Biphasic scaffold and compact layer porosity and water absorptivity were determined by previously reported methods [33], [35].

A suspension of 0.5 ml BMSCs was administered to the surface of compact layer at 37°C in an atmosphere of 5% CO_2_ and 95% humidity. After 8 h, the amount of fluid and cells permeating through the compact layer was observed.

### Biomechanical Assay of Biphasic Scaffolds

A computer-controlled electronic universal material testing machine (Shimadzu, Kyoto, Japan) was used for biomechanical testing. Both ends (approximately 1 mm) of the cell-free biphasic scaffold were embedded in block of polymethylmethacrylate (PMMA) to provide gripping points for biomechanical testing. Observation of 80% breakage at phase juncture was considered failure. Results were the average of 6 independent measurements per sample, expressed as mean±standard deviation (mean ± STD). The investigator performing the mechanical testing was blind to the type of biphasic scaffold.

Biphasic scaffolds were subjected to shear testing at speed of 1 mm/min until failure. The maximal shear strength was calculated as maximal measured force (N) per cross-sectional area (m^2^). Similarly, biphasic scaffolds were subjected to tensile testing by pulling along the longitudinal axis at a constant speed of 1 mm/min until failure. The maximal tensile strength was calculated as the maximum tensile strength (N) per cross-sectional area (m^2^).

### BMSCs Isolation, Culture, Proliferation, and Induction

In this study, all experimental procedures involving animals were approved by the Animal Care and Experiment Committee of Fourth Military Medical University. BMSCs were obtained from the iliac crests of New Zealand white rabbits (more than 24 months old, *n = *20), as described previously [36], and cultured to 80% confluence. Third passage BMSCs were cultured in chondrogenic or osteogenic induction medium, respectively. Chondrogenic induction medium contained low DMEM, 10 ng/ml TGF-β_2_ (Pepro Tech, Rocky Hill, NJ, USA), 1% ITS^+^ premix (BD, Franklin Lakes, NJ, USA), 10^−7^ M dexamethasone (Sigma, USA), 50 µg/ml ascorbic acid (Sigma, USA), 1 mM sodium pyruvate (Sigma, USA), 4 mM proline (Sigma, USA), and 1% antibiotics solution [31]. The osteogenic induction medium contained high DMEM, 10% FBS, 10^−8^ mol/L DEX, 10 mM β-glycerol phosphate, and 50 mg/ml L-ascorbic acid) [32]. The medium was replaced every three days and the cells were harvested at day 21 for seeding. By day 21 following induction, both chondrogenic and osteogenic induced BMSCs were demonstrated respectively.

### In Vitro Proliferation and Morphology of Induced BMSCs on Biphasic Scaffolds

Chondrogenic and osteogenic induced BMSCs were resuspended in culture medium (cell density, 1×10^7^ cells/ml), respectively. Chondrogenic induced BMSCs were seeded onto the chondral phase (2×10^5^ cells/scaffold), and osteogenic induced BMSCs were seeded onto bony phase (5×10^5^ cells/scaffold). The seeding method is shown in Fig. S1. Cells were allowed to attach to the scaffolds for 2 h at 37°C prior to the addition of fresh DMEM containing 10% FBS and 1% antibiotics solution. The hybrid experimental and control biphasic scaffolds were then further incubated at 37°C in an atmosphere of 5% CO_2_ and 95% humidity for three days. Adhesion properties, morphological characteristics,and cellular distribution within scaffolds were analyzed by SEM (S-3400N, Hitachi, Tokyo, Japan).

### Surgery

**Figure 2:**
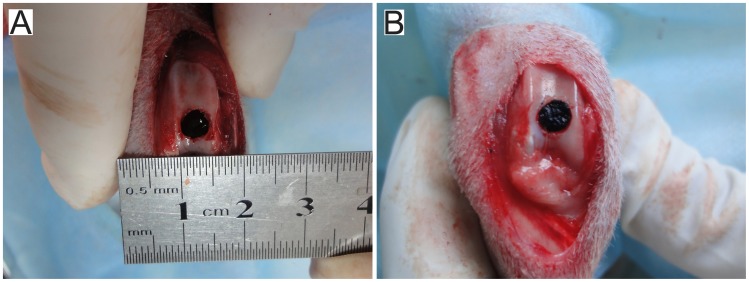
Implantation surgery. (A) An osteochondral defect (diameter = 5 mm; depth = 6 mm) generated in the rabbit knee patellofemoral groove surface. (B) Prepared autogeneic tissue-engineered osteochondral composite inserted into the defect, and the surface of the composite flushed with the articular surface.

After approximately 4 weeks, the rabbits were again anesthetized. A medial para-patellar skin incision was made, allowing exposure of the knee joint via lateral dislocation of the patella. A full-thickness cylindrical osteochondral defect was created in the patellofemoral groove with 5 mm diameter and 6 mm depth using a surgical drill (Fig. 2A). During surgery, the prepared autogeneic tissue-engineered osteochondral composites were carefully inserted into the defects so that the composite surface was flushed with the articular surface (Fig. 2B). In each subject, the left and right knee joints were administered with experimental and control type scaffolds, respectively. Thus, 40 defects in 20 rabbits were assigned evenly to two treatment groups. Additionally, 6 New Zealand white rabbits (more than 24 months old) were assigned to the non-treated group. The weight and age of the rabbits were similar to those in the experimental group and the control group. All rabbits (n = 26) in this study were randomly assigned to the three groups and were raised in the same environment. The non-treated group was subjected to the same defects of the patellofemoral grooves; however, no scaffold was implanted. After implantation, the knee joint synovium, capsule and skin were separately sutured. All rabbits subjects resumed standing activity on all four legs immediately following recovery from anesthesia.

### Macroscopic Examination

At 3 and 6 months after surgical implantation of the biphasic scaffolds, 10 rabbits (10 experimental knees; 10 control knees) and 3 non-treated rabbits (6 non-treated knees) were killed via pentobarbital overdose. The knee joints were exposed and neocartilage was examined macroscopically for scoring using a previously developed scale [37]. Briefly, score were based on the coverage, color, and surface of cartilage and defect margins. Following macroscopic examination, half of the samples of experimental and control groups underwent Micro-CT scanning followed by histological and immunohistochemical examination. Remaining samples underwent biomechanical and biochemical analysis.

### Micro-CT Scanning

Micro-CT scanning was performed to quantitatively evaluate regeneration of the osteochondral defects of experimental and control groups. Rabbit femur ends fixed in 4%-paraformaldehyde were placed on a sample holder with the femur axis oriented perpendicular to the scanning plane. Samples were scanned at 45 µm voxel resolution. 3-D isosurface renderings were regenerated by Microview v1.1.1 software (GE Medical Systems) to visualize the regenerated osteochondral tissue. From the reconstruction data, a cuboid region of interest (ROI) was selected for analysis corresponding to the original defect location. The degree of cartilage and bone regeneration within the osteochondral defect and bone mineral density (BMD) of subchondral bone were observed. Moreover, a cylindrical region of interest (ROI) (diameter = 4.5 mm) was selected to analyze the volume of the neocartilage corresponding to the original defect location. The image threshold of the cartilage was regarded as 0 to 200Hu. Before the cartilage was measured, the subchondral bone was removed out of the ROI and the cartilage was selected. With this method, only the cartilage phase was ensured to be measured.

### Histological and Immunohistochemical Evaluation

Following micro-CT evaluation, specimens were decalcified in ethylenediamine tetra-acetic acid (EDTA) solution, dehydrated in a graded series of acetone, and embedded in paraffin. Some sections (thickness = 5 µm) were stained with hematoxylin and eosin (H&E). Some sections were decerated through xylene followed by graded ethyl alcohols progressing to tap water. For Toluidine blue staining analysis, after being stained with 0.04% Toluidine blue for 5 to 10 minutes, they were then rinsed in tap water and dried in warm air. For Safranin O staining analysis, sections were stained with Weigert’s hematoxylin solution for 10 minutes, and rinsed in running tap water. Then they were placed in fast green for 5 minutes, quickly rinsed in 1% acetic acid solution, stained with 0.1% Safranin O solution for 5 minutes, and dehydrated in 100% alcohol. For immunohistochemical analysis, sections were deparaffinized with xylene, rehydrated in a graded ethanol series and washed with PBS. Type II collagen was detected immunohistochemically using monoclonal antibodies (433120; Invitrogen, Camarillo, CA, USA) as previously described [38]. Regenerated osteochondral tissue was scored using the ICRS Visual Histological Assessment Scale [39]. This scoring system was primarily based on the surface, matrix, cell distribution, cell population viability, subchondral bone, and cartilage mineralization. Specimens were independently scored by two evaluators who were blinded to clinical data. A representative score for each parameter was determined by averaging the scores of the two observers.

### Biochemical Assay of Neocartilage

Cylindrical-shaped regenerated osteochondral tissues of 4.5 mm diameter and 5–8 mm depth were taken from the patellofemoral grooves at 3 and 6 months after implantation. The neocartilage (mean thickness = 0.75 mm) was obtained when the subchondral bone was ablated under an operating microscope. In addition, the cylindrical-shaped native cartilage samples 4.5 mm in diameter obtained from the patellofemoral grooves of 4 healthy New Zealand white rabbits were tested as a normal control. The rabbits’ weights and ages were similar to those in the three groups. The thickness of tissues tested, and method for determining such thickness are shown in Fig. S2. The mean wet weight of the neocartilage and native cartilage is shown in Table S1. The photographs showing the measurement of the wet weight of some neocartilage are shown in Fig. S3. GAG and collagen contents of the neocartilage were analyzed, as described by Jia et al [31]. Briefly, the samples were digested in lysis buffer containing 125 µg/ml papain (Sigma, USA), 10 mM cysteine hydrochloride (Sigma, USA), 100 mM sodium phosphate, and 10 mM EDTA, for 24 h at 60°C [40]. GAG content was quantified using a Blyscan™ Sulfated Glycosaminoglycan Assay kit (Biocolor, Carrickfergus, Northern Ireland, UK) and a spectrophotometer (Bio-Tek Instruments, Winooski, VT, USA) at 656 nm, using dimethylmethylene blue binding [41]. A standard curve was established using bovine cartilage chondroitin sulfate (Sigma, USA) [42]. Aliquots of the papain-digested sample solution were incubated for 48 h at 48°C in 0.05 M acetic acid containing 0.5 M NaCl (pH 3) and 10 mg/ml pepsin (Sigma, USA). Collagen content was determined by the Sircol™ Soluble Collagen Assay kit (Biocolor) with absorbance measured by spectrophotometer (BioTek) at 555 nm. A standard curve was generated using L-hydroxyproline (Sigma, USA) [43].

### Statistical Analysis

Statistical analysis was performed using the SPSS 13.0 software package. All values were expressed as mean±standard deviation (STD). Data from the experimental group, the control group, the non-treated group and native cartilage were analyzed statistically. A 1-way ANOVA, repeated measurement ANOVA and T-test were used respectively. *P*-values of less than 0.05 (*P*<0.05) were considered statistically significant.

## Results

### Characterization of the Compact Layer

**Figure 3:**
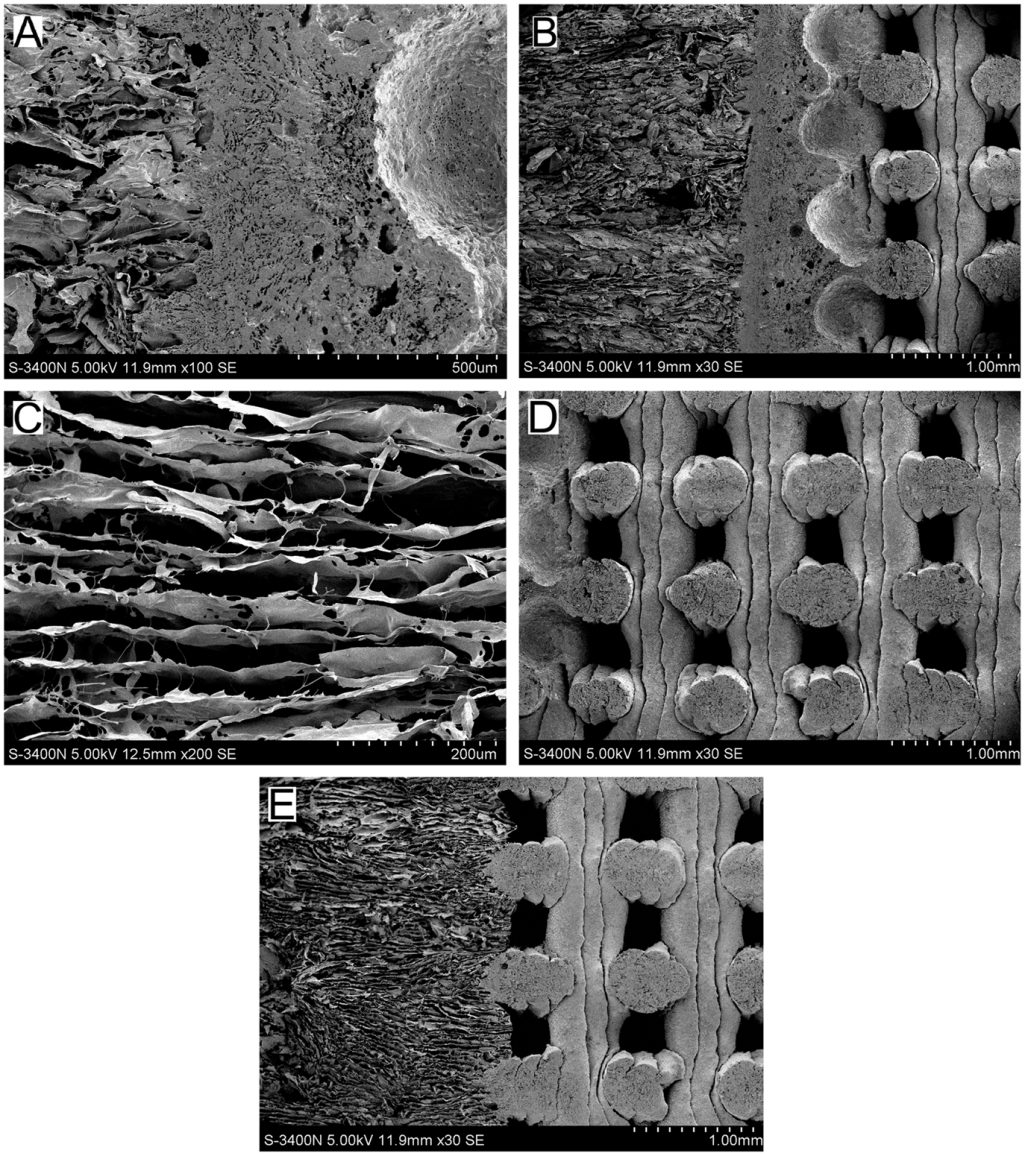
SEM micrographs showing the longitudinal section of the biphasic scaffold. (A) the compact layer. (B) the compact layer-containing biphasic scaffold. (C) the chondral phase. (D) the bony phase. (E) the compact layer-free biphasic scaffold.

A longitudinal section of the compact layer-containing biphasic scaffold (experimental group) is shown in Fig. 3A. The middle region of the SEM image shows the magnified compact layer composed of PLGA/β-TCP. The compact layer is demonstrated to form a composite of the two different phase scaffold materials. Numerous micropores were observed in the compact layer (diameter = 9.2±3.3 µm). Independent micropores were not observed to form microtubules.

The water absorption capacity was mere 2.8±0.2%, and the porosity ratio was 3.2±0.5%. After the addition of 0.5 ml BMSCs suspension on the compact layer surface, no fluid and cell permeated through the compact layer, indicating a waterproof and hydrophobic compact layer.

### Characterization of Biphasic Scaffold

The compact layer-containing biphasic scaffold was separated by the compact layer, as shown in Fig. 3B. In the longitudinal section (Fig. 3C), the oriented structure of the chondral phase appears microtubule-like, with interconnected tubules in a parallel arrangement. Fig. 3D, an image at low magnification, demonstrated the bony phase fabrication by clearly showing the consecutively overlapping trilayers in the desiccated specimen. The transverse section revealed pores of approximately 350×350 µm^2^ in size. The microstructure of the compact layer-free biphasic scaffold was shown in Fig. 3E, further demonstrating the direct connection between the chondral and bony phases. Notably, about half of oriented collagen was observed to insert into the bony phase, while the remained half was observed to be unconnected.

In the present study, the experimental and control group were all of the same size. The observed height of the chondral and bony phase was 2 mm and 4 mm, respectively (Fig. 3A). No significant difference were observed in the porosity of the compact layer-containing and compact layer-free biphasic scaffold types (88.6%±3.3% and 89.9%±3.7% respectively).

### Proliferation of Induced BMSCs on Biphasic Scaffolds

**Figure 4:**
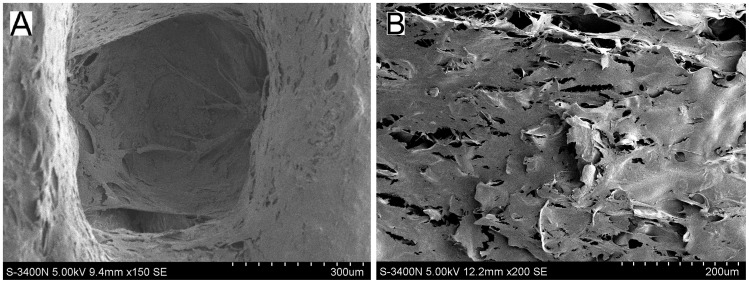
SEM micrographs showing adhesion and morphology of the induced BMSCs cultured on the chondral and bony phase. (A) Osteogenic-induced BMSCs on the bony phase. (B) Chondrogenic-induced BMSCs on the chondral phase.

SEM micrographs showing adhesion and cell morphology on biphasic scaffolds are presented in Fig. 4. Good distribution and proliferation of BMSCs on the biphasic scaffolds were observed after culture for 7 days. Cells were observed to cluster on the surface and extend into the pores. Additionally, cell pseudopodium appeared stretched and tightly attached to scaffold surface. These observations indicated the hydrophilic nature of the biphasic scaffolds, an optimal environment for adherence and proliferation of induced BMSCs.

### Biomechanical Assay of Biphasic Scaffolds

**Figure 5:**
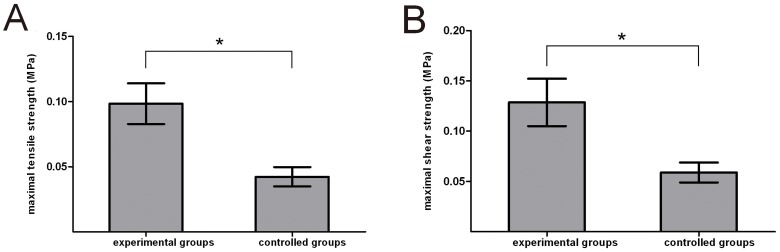
Biomechanical properties of cell free-biphasic scaffolds in experimental and control groups. (A) Maximal tensile strength; (B) Maximal shear strength. *p<0.05, significance between the two groups.

The *in vitro* biomechanical properties of the two biphasic scaffold types are detailed in Fig. 5. The maximal tensile and shear strength of the experimental group was superior to the control group (*P<*0.05).

### Macroscopic Evaluation of Neocartilage Surface

No evidence of inflammatory response, disability, or deterioration was observed in any of the joints. In the control group, 5 implanted compact layer-free biphasic scaffolds were detached and chondral phases were taken into the articular cavity by joint motion. The defects were completely occupied by fibrous tissues, with dents clearly evident on the surface. In experimental group, no osteochondral composites detachment was founded.

**Figure 6:**
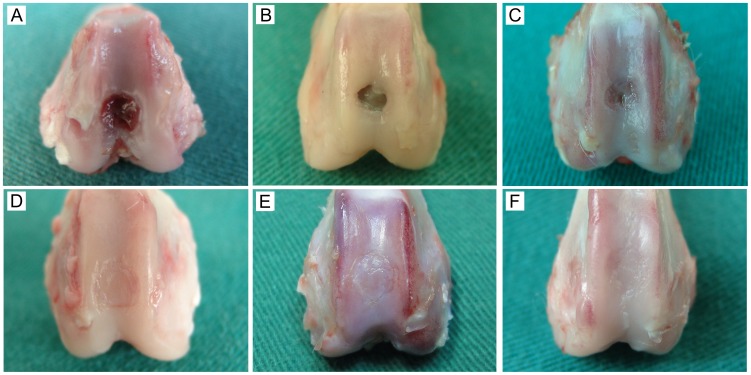
Macroscopic appearance of the repaired cartilage. (A) Non-treatment group specimen at 3 months after surgery. (B) Non-treatment group specimen at 6 months. (C) Control group specimen at 3 months. (D) Control group specimen at 6 months. (E). Experimental group specimen at 3 months. (F) Experimental group specimen at 6 months.

All defects in non-treatment group were occupied by necrotic tissue and sparse fibrous tissue by 3 months after surgery. In some cases, cartilage-like tissue was observed at the periphery. At postsurgical months 3, the 7 examined defects in the control group evidenced repair by thin layers of rough hyaline cartilage, with a coverage rate of about 25%. Notably, defect surfaces were visibly lower than adjacent native cartilage surfaces (Fig. 6A). In the experimental group, the defects were covered by neocartilage with a relatively smooth surface. Additionally, the rates of coverage in all defects were 100%. Though the surface of neocartilage was flush with the adjacent native cartilage, a visible border was apparent between native and restored tissue (Fig. 6C).

By 6 months after surgery, obvious pits were apparent at the defects in the non-treatment group and repair consisted primarily of bulky fibrous tissue. The rest 8 defects in the control group evidenced restoration with a rough neocartilage surface and a coverage rate of more than 75%. The neocartilage thickness was uneven, and the neocartilage surface remained lower than adjacent native cartilage. In addition, distinguishable fissures in the neocartilage were observed in 4 defects (Fig. 6B). In the experimental group, the neocartilage appeared macroscopically similar to the native cartilage, and the neocartilage surface was flush with the adjacent native cartilage (Fig. 6D).

**Figure 7:**
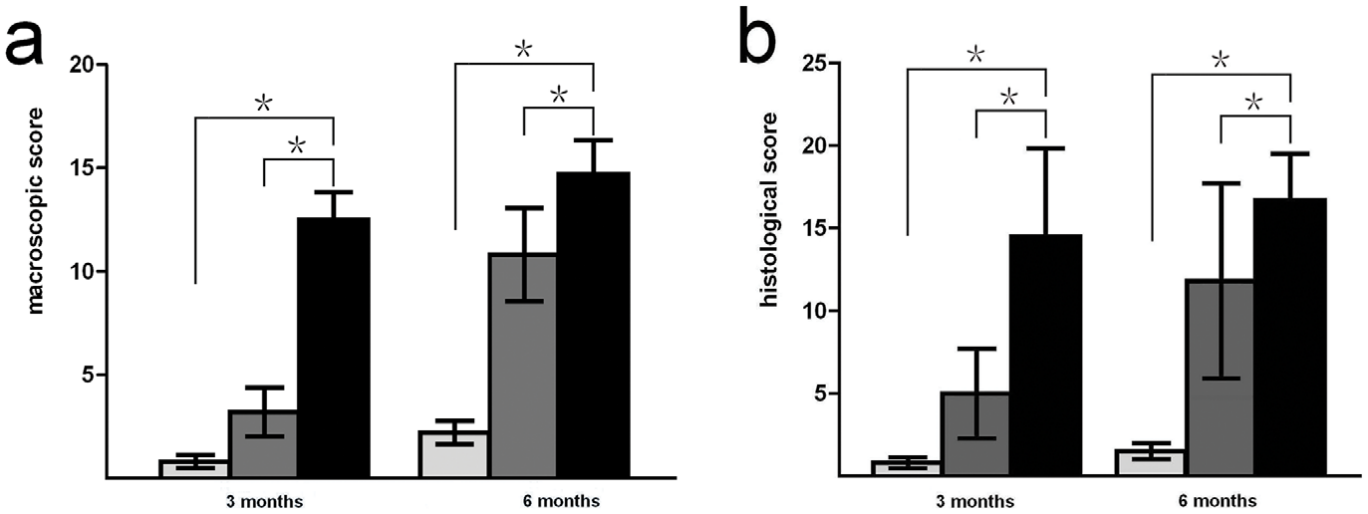
Macroscopic evaluation scoring for neocartilage surface and histological evaluation of regenerated osteochondral tissues. (A) Macroscopic score for neocartilage surface. (B) Histological score for regenerated osteochondral tissue. At 3 and 6 months after implantation, experimental group scores were significantly higher than both control and non-treatment group scores (*P<*0.05). (Black bars represent the experimental group, gray bars represent the control group, light gray bars represent the non-treated group. *p<0.05, significance between the two groups.).

The scoring for the experimental, control, and non-treatment groups is detailed in Fig. 7A. The macroscopic scores obtained from the experimental group were statistically superior to scores for both the control and non-treated groups [3 months: experimental versus non-treatment group (*P* = 0.000), experimental versus control group (*P* = 0.000); 6 months: experimental versus non-treatment group (*P* = 0.000), experimental versus control group (*P* = 0.001)].

### Micro-CT Evaluation

Due to the lack of neocartilage for testing, no data was generated for the non-treatment group. Both the experimental and control groups were evaluated by Micro-CT scanning. Each ROI was selected according to the original osteochondral defect location.

**Figure 8:**
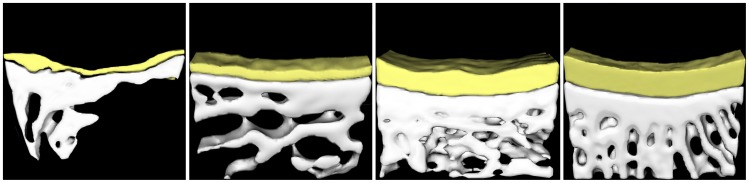
Micro-CT 3-D reconstruction image. (A) Control group defect at 3 months after surgery. (B) Control group defect at 6 months. (C) Experimental group defect at 3 months. (D) Experimental group defect at 6 months.

At 3 months after surgery, control group defects were repaired by thin layers of cartilage and sparse, irregular osteotylus. The surface of the cartilage was rough and little subchondral bone was formed (Fig. 8A). In the experimental group, though the thickness of neocartilage was equal to that of native cartilage, the surface was not smooth. The regenerated bone trabecula structure was highly irregular, forming in a non-vertical orientation relative to the articular surface (Fig. 8C).

By 6 months following surgery, the defects in the control group exhibited superior regeneration than observed at the 3 month examination. The neocartilage surface was uneven and remained thin; however, significant amounts of non-oriented bone trabecula were observed under the neo-cartilage (Fig. 8B). The experimental group exhibited repaired tissues with imageological features similar to those of the native articular tissue. The neocartilage surface was smooth and uniform, and the neocartilage thickness indistinguishable from native cartilage. Additionally, regenerated bone trabecula were arranged in a vertical orientation relative to the articular surface (Fig. 8D).

**Figure 9:**
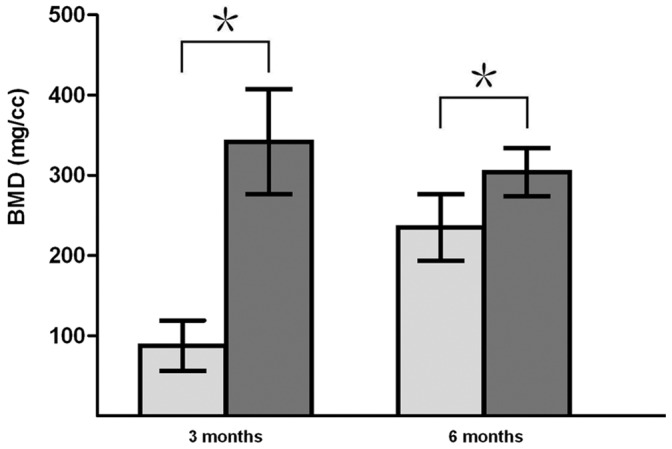
BMD results for newly-formed subchondral bone quantified by Micro-CT. At 3 and 6 months following biphasic scaffold implantation, significant differences were observed between the experimental and control groups (*P<*0.05). (Gray bars represent experimental group, light gray bars represent control group. * *P<*0.05, significance between the two groups.).

The subchondral bone BMDs of the experimental and control groups are shown in Fig. 9. At 3 months and 6 months, respectively, subchondral bones of the experimental group exhibited higher BMDs (341.65±65.34 mm^3^ and 47.75±6.76 mm^3^) than those of the control group (29.12±6.18 mm^3^ and 41.54±6.24 mm^3^) (*P<*0.05). Due to progressive reconstruction, the subchondral bone BMD of the experimental group at 3 months was significantly higher than the BMD observed at 6 months.

From the reconstruction data, a cylindrical region of interest (ROI) (diameter = 4.5 mm) was selected to analyze the volume of the neocartilage corresponding to the original defect location. The volume of the neocartilage is shown in Fig. S4. The representative images of the regenerated tissue are shown in Fig. S5.

In agreement with the macroscopic evaluation, the experimental group evidenced superior results by micro-CT scanning than those observed in the control group.

### Histological Evaluation

**Figure 10:**
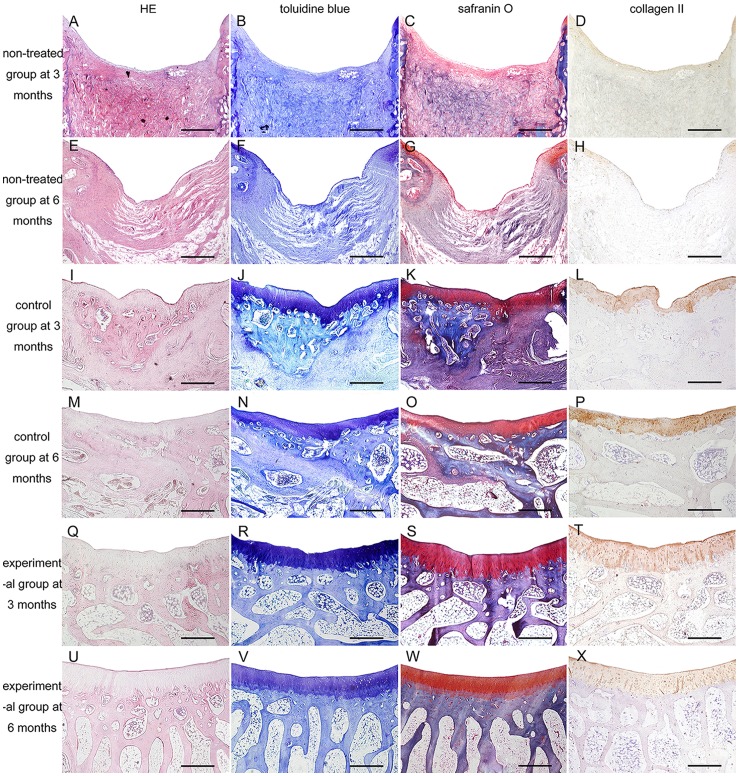
Histological and immunohistochemical analyses of repaired osteochondral tissue. (A,E,I,M,Q,U) H&E staining. (B,F,J,N,R,V) Toluidine blue staining. (C,G,K,O,S,W) Safranin O staining. (D,H,L,P,T,X) Immunohistochemical staining for collagen type II. (A,B,C,D) Non-treated group defects at 3 months after surgery. (E,F,G,H) Non-treated group defects at 6 months. (I,J,K,L) Control group defects at 3 months. (M,N,O,P) Control group defects at 6 months. (Q,R,S,T) Experimental group defects at 3 months. (U,V,W,X) Experimental group defects at 6 months. Scale bars = 1 mm.

All histological specimens were harvested successfully, and some regenerated osteochondral tissues were subjected to HE staining (Fig. 10A–D). Toluidine blue, Safranin O, and immunohistochemical staining were used to confirm the presence of Type II collagen in the neocartilage (Fig. 10E–P). Within 3 months of implantation, all biphasic scaffold materials were completely absorbed by regenerated osteochondral tissues, and no evidence of deterioration was observed sampling any specimen. Supporting macroscopic findings, the experimental group evidenced superior histological characteristics than those observed in the control and non-treatment groups.

By 3 months after surgery, defects in the non-treatment group were occupied by bulky necrotic tissue and sparse fibrous tissue. The control group evidenced repair by regenerated hyaline cartilage with visible fissures. Some neocartilage cells were organized in clusters, and neocartilage layers were generally very thin. Under the hyaline cartilage, sparse and irregular osteotylus were present along with bulky fibrous tissue. Furthermore, no evidence of bone trabecula formation was observed (Fig. 10A,E,I,M). In experimental group, the regenerated chondrocytes exhibited a columnar orientation. Though the neocartilage thickness resembled native cartilage, the surface was not very smooth. Regenerated subchondral bone exhibited highly irregular bone trabecula structures in a non-vertical orientation relative to the articular surface (Fig. 10C,G,K,O).

By 6 months after surgery, defects in the non-treatment group were completely replaced by fibrous tissue. In the periphery of the defects, sparse regions of newly-formed osteochondral tissue were observed. At the locations of the original defects, deep pits remained visible. Defects in the control group were observed to have much greater regeneration than observed at 3 months after surgery. The neocartilage surface remained uneven and thin, though fibrous tissue was largely replaced by irregular bone trabecula. Notably, no oriented structure was present in the regenerated subchondral bone (Fig. 10B,F,J,N). In the experimental group, repaired tissues presented histological features were similar to those of native articular tissue, including a smooth and uniform neocartilage surface, round chondrocytes with cartilage lacunae distributed in columns and aligned perpendicular to the articular surface, and bone trabecula arranged vertically relative to the articular surface. Furthermore, visible fusion of neocartilage with the regenerated subchondral bone was observed with a clear tidemark (Fig. 10D,H,L,P).

According to histological grading, experimental group scores were all significantly superior to those of both the control and non-treatment groups [3 months: experimental versus non-treatment group (*P* = 0.000), experimental versus control group (*P* = 0.000); 6 months: experimental versus the non-treatment group (*P* = 0.000), experimental versus control group, (*P = *0.042)] (Fig. 7B).

### Biochemical Assay of Neocartilage

**Figure 11:**
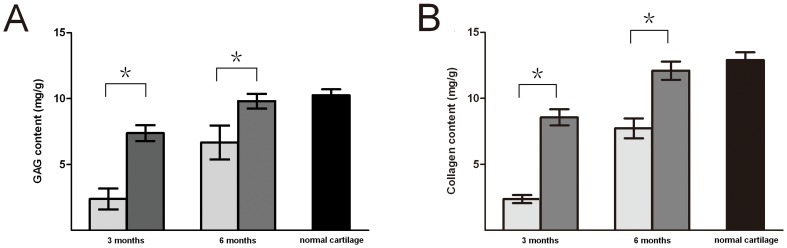
Biochemical characterization of cartilage. Contents of GAG (A) and collagen (B) showed a significant difference between the experimental and control groups (*p<*0.05). At 3 months after implantation, the neocartilage in the experimental group differed significantly from the native cartilage (*P<*0.05). At 6 months, however, no significant differences were observed (*P*>0.05). (Gray bars represent cartilage in experimental group, light gray bars represent cartilage in control group and black bar represents native cartilage. * *P<*0.05, significance between the two groups. ).

Due to the lack of neocartilage for testing, no data was generated for the non-treatment group. The results of biochemical characterization of the experimental and control group neocartilage specimens at 3 and 6 months following implantation versus native cartilage were shown in Fig. 11. The GAG and collagen contents in both groups of neocartilages increased in a time-dependent manner. GAG and collagen contents were similar in the two experimental groups at 3 months and 6 months. At 3 months, GAG and collagen in the two groups were all much lower than those of native cartilage (*P<*0.05). While at 6 months, there were no significant difference between experimental group and native cartilage (*p>*0.05).

## Discussion

In this study, we successfully developed a novel biphasic scaffold with two phases connected by a compact layer interface for applications in osteochondral tissue repair. The compact layer was shown to facilitate superior integration between the chondral and bony phases, thus significantly enhancing the biomechanical properties of the biphasic scaffold. Compact layer-containing biphasic scaffolds were seeded with chondrogenic and osteogenic induced BMSCs and implanted into osteochondral defects in a rabbit knee model. After 6 months, regenerated osteochondral tissues with good function were observed in this experimental group. *In vivo* experiments further demonstrated that the optimum environments provided by the compact layer facilitated induced BMSCs proliferation and differentiation, thus meeting requirements for clinically viable levels of osteochondral regeneration.

The compact layer was fabricated on the top of the bony phase using rapid prototyping technologies [32]. By using the dissolving-conglutination method, the compact layer facilitated the integration between the chondral and bony phases. This procedure was easier than other strategies [44]–[48]. The biomechanical experiments were employed to provide quantitative assessment of the integration strength between these two phases with and without the compact layer [49]. The results revealed that maximum tensile strength and shear strength in the experimental group were 2.32-fold and 2.17-fold higher, respectively, than those of the control group. Thus, it could be concluded that the presence of a compact layer joining the two phases dramatically enhanced the mechanical properties of the biphasic scaffold. After implantation into the osteochondral defect site, bipasic scaffold must bear considerable mechanical load generated by typical joint motion in order to maintain joint integrity and provide long-term fixation of the recipient site. *In vivo* experiment demonstrated that 5 from 20 tissue-engineered osteochondral composites in the control group evidenced detachment, with chondral phases dropping into the articular cavity. Conversely, no osteochondral composites in the experimental group showed signs of detachment. These results indicated that the compact layer-containing biphasic scaffolds are better suited to meet the mechanical demands of the joints. More importantly, these findings also demonstrated that the compact layer played a critical role in promoting the biomechanical property of the bipasic scaffold.

By analyzing the SEM images, those phenomena could be well explained. In the compact layer-containing biphasic scaffolds, the compact layer and bony phase were fused completely and a wall-like oriented collagen was completely inseted into the compact layer. In the compact layer-free phasic scaffolds, the bony and chondral phases exhibited a direct connection with much less structural integrity. About 50% of oriented collagen was inserted into the bony phase, while about 50% was unconnected. Thus, mechanical properties in the bonding region of control biphasic scaffolds were much inferior to those observed in experimental biphasic scaffolds.

Articular cartilage and subchondral bone require different environments for cell proliferation and growth. These unique environments can be separated and maintained by applying biphasic scaffolds containing a compact layer. Articular cartilage derives oxygen and nutrient primarily from the synovial fluid, where oxygen partial pressure and pH are much lower than in vascularized subchondral bone [22], [50]. Some studies have substantiated that chondrocytes are well adapted to a hypoxic environment, and that culturing in a low O_2_ tension environment may be beneficial to speedy recovery of the differentiated phenotype [51], [52]. Once blood infiltrates the articular cavity, the antigenic products of leukocytes may be released into the synovial fluid, provoking the release of inflammatory mediators that cause necrosis in new chondrocytes [22]. Calcified layer can resists vascular invasion from subchondral tissue and prevent osseous upgrowth. This barrier is essential for maintaining the integrity of the repaired cartilage [23]–[26]. Moreover, the synovial fluid is detrimental to osteogenic activity [26]. With this barrier, the impediments to the newly-born osteocytes posed by the synovial fluid can be overcome.

In the present study, a biphasic scaffold mimicking natural articular osteochondral structure was developed. Previous studies had shown that bony and chondral phase scaffolds provide optimal platforms for growth of induced BMSCs both *in vitro* and *in vivo.* These biphasic scaffolds promote the adherence, distribution, proliferation, and differentiation of BMSCs [31], [32]. The addition of a compact layer between the two phases of the biphasic scaffold was expected to further improve proliferation and differentiation of induced BMSCs in the recipient site. The compact layer acted as a calcified layer between chondral and bony biphases. Numerous micropores with diameters less than 10 µm were observed in the compact layer via SEM. These micropores were independent, and no evidence of microtubule formation was observed. *In vitro* examination of water absorption capacity and porosity ratio in using biphasic scaffolds possessing a compact layer revealed that both values were lower than those reported by previous studies of bony or cartilage scaffolds. Additionally, in the *in vitro* experiment, fluid and BMSCs could hardly permeate through the compact layer with a 0.5 mm thickness. These results indicated that the compact layer was hydrophobic and waterproof. Prior to natural biodegradation of the scaffold *in vivo*, the compact layer may thus inhibit fluid infiltration during early stages of healing, providing chondrocytes and osteocytes optimal and independent environments for regeneration during this critical time. Accompanied by degradation of the compact layer, the newly-born chondrocytes and osteocytes began to produce extracellular matrix to form native calcified layer. Then the independent environment would be sustained, and avoid being impaired.

Upon examination of *in vivo* subjects at 3 and 6 months, chondral tissue regeneration in the experimental group and control group were compared by examining Micro-CT images, gross morphology, histological and immunohistochemical characteristics, and biochemical functions. These results indicated that regenerated chondral tissues of the experimental group were more abundant and more closely resembled native tissues than regenerated chondral tissues of the control group. Overall, *in vivo* results indicated that the compact-layer method proposed in the present studies represented a substantial advance in cartilage repair over conventional methods. Histological and immunohistochemical results further confirmed that the experimental group exhibited superior chondrocyte proliferation and columnar orientation by 6 months after implantation. Furthermore, neocartilage thickness was also virtually identical to thicknesses observed in native cartilage, and a well-shaped bone trabecula was observed in an orientation vertical to the surface of joint. These observations provide a good explanation for elevated neocartilage GAG and collagen contents, and superior gross morphology results observed in the experimental group. The success of the compact layer was due to good proliferation and differentiation of the induced BMSCs in the compact layer-containing biphasic scaffold in the recipient site.

However, our investigation had certain limitations: the biphasic scaffold was not combined with growth factor delivery system, which would promote proliferation and differentiation of the induced BMSCs [53]. A deep drill hole defect was not an ideal control as a standard method of treatment. Due to our lack of experience and the specifics of the assay kits, the values for GAG and collagen appear to be off by several fold to approximately an order of magnitude. Even in the control group, staining appeared quite strong. The staining intensity of the sections could not accurately represent the quality of neocartilage. Only the morphology of newly formed cartilage and subchondral bone were worth observing. Hence, these work requires further efforts in future. In the current study, we did not observe the phenomenon of neocartilage deterioration in any specimens. In additional studies, prolonged timeframes will be applied to further observe. Furthermore, the defect was in a region of the rabbit joint that was not representative of the load-bearing femoral condyles. However, because the femoral condyles of rabbits were too small to create defects with a 5 mm diameter, the patellofemoral grooves were used due to their larger size and greater accessibility in the rabbit model. In future studies, the femoral condyles of larger animal models, such as pigs or sheep, will be used. In biomechanical assay of biphasic scaffolds, the strain was distributed across cartilage phase, bone phase and compact layer. Since they had different tensile properties, the interpretation of comparisions among groups was somewhat complicated.

In summary, the compact layer-containing biphasic scaffold exhibited superior biomechanical properties in *in vitro* studies. In addition, the biomimical compact layer-containing biphasic scaffold combined with chondrogenic and osteogenic induced BMSCs yielded excellent functional regenerated osteochondral tissues *in vivo*. These findings demonstrated that addition of a compact layer to biphasic scaffolds enhances integration and also provides chondrogenic and osteogenic induced BMSCs with independent environments optimal for proliferation and differentiation. These unique features indicate the potential clinical application of this strategy for osteochondral tissue engineering.

## Supporting Information

Figure S1 **The method about seeding cells into the cartilage and bone phases.** A cartilage culture chamber with a hole of 5 mm diameter was made using the anterior portion of a 20 ml syringe. The compact layer was wrapped in medical coated fabric, and the biphasic scaffold was fixed at the hole. Then, the cartilage culture chamber was place in the culture capsule. Thus, the two phases were provided with independent culture environments. After the biphasic scaffolds and culture capsules were sterilized by exposure to 20 kGy 60Co radiation, the two cell suspensions were repeatedly dropped onto the chondral and bony phases. Static seeding methods were then employed in this study.
(TIF)

Figure S2 **The thickness of the neocartilage.** The mean thickness was approximately 0.75 mm. Two methods were used involving (i) the gross appearance and (ii) the histological section (bar = 1 mm).
(TIF)

Figure S3 **The measurement of the wet weight of some neocartilage.**
(TIF)

Figure S4 **The volume of the neocartilage.** The reconstruction utility was used to quantify the volume of the neocartilage. From the reconstruction data, a cylindrical region of interest (ROI) (diameter = 4.5 mm) was selected to analyze the volume of the neocartilage corresponding to the original defect location. (Gray bars represent experimental group, light gray bars represent control group.)
(TIF)

Figure S5 **The representative images of the regenerated tissues.**
(TIF)

Table S1 **The mean wet weight of the neocartilage and native cartilage (diameter = 4.5 mm).**
(TIF)
