# Supplementary figures and images for: The Impact of Compact Layer in Biphasic Scaffold on Osteochondral Tissue Engineering
Source: PLoS One. 2013 Jan 28;8(1):e54838. doi: 10.1371/journal.pone.0054838 (PMC3557302; doi:10.1371/journal.pone.0054838)

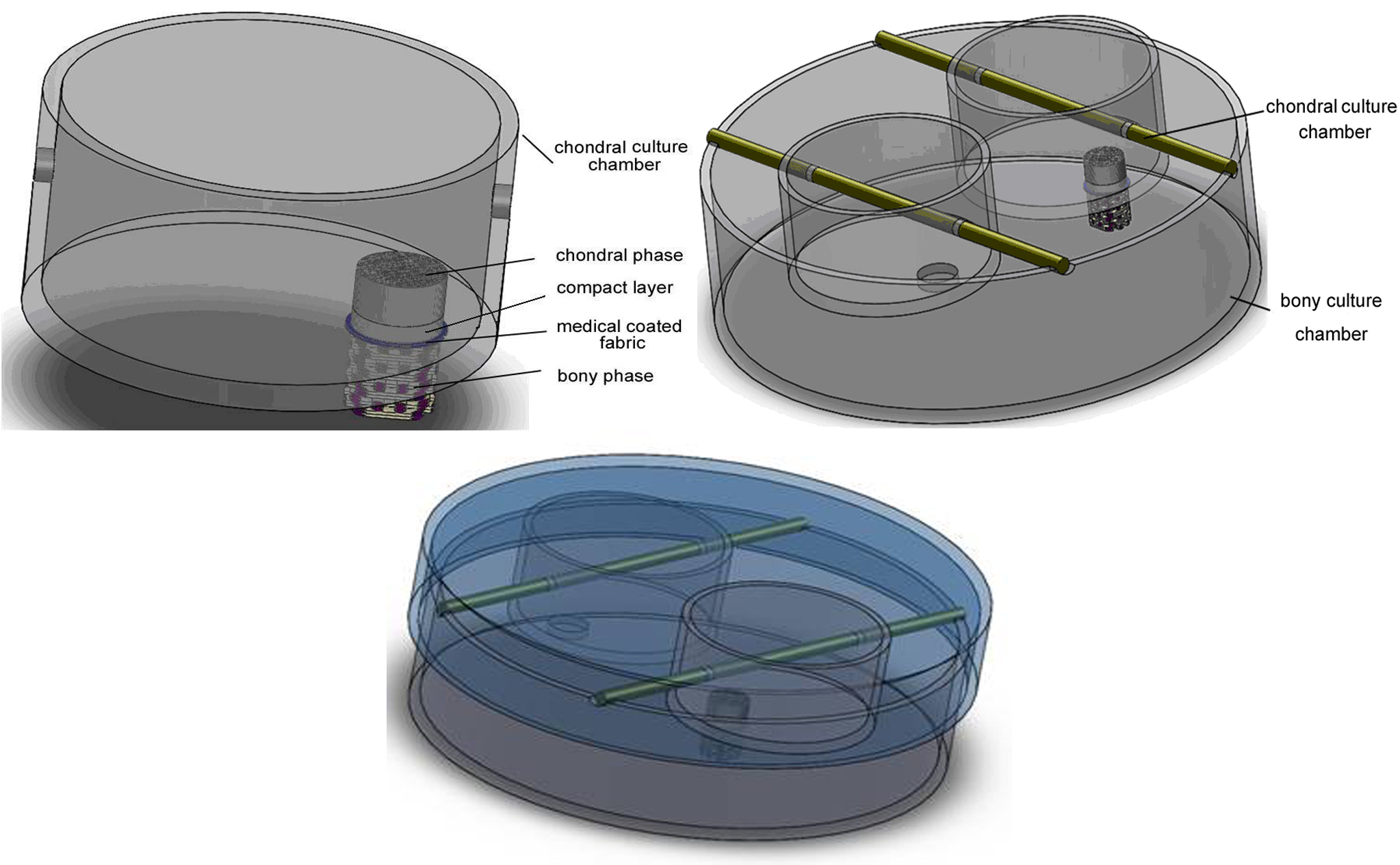

Supplement: Figure S1 — The method about seeding cells into the cartilage and bone phases. A cartilage culture chamber with a hole of 5 mm diameter was made using the anterior portion of a 20 ml syringe. The compact layer was wrapped in medical coated fabric, and the biphasic scaffold was fixed at the hole. Then, the cartilage culture chamber was place in the culture capsule. Thus, the two phases were provided with independent culture environments. After the biphasic scaffolds and culture capsules were sterilized by exposure to 20 kGy 60Co radiation, the two cell suspensions were repeatedly dropped onto the chondral and bony phases. Static seeding methods were then employed in this study. (TIF) [file pone.0054838.s001.tif]

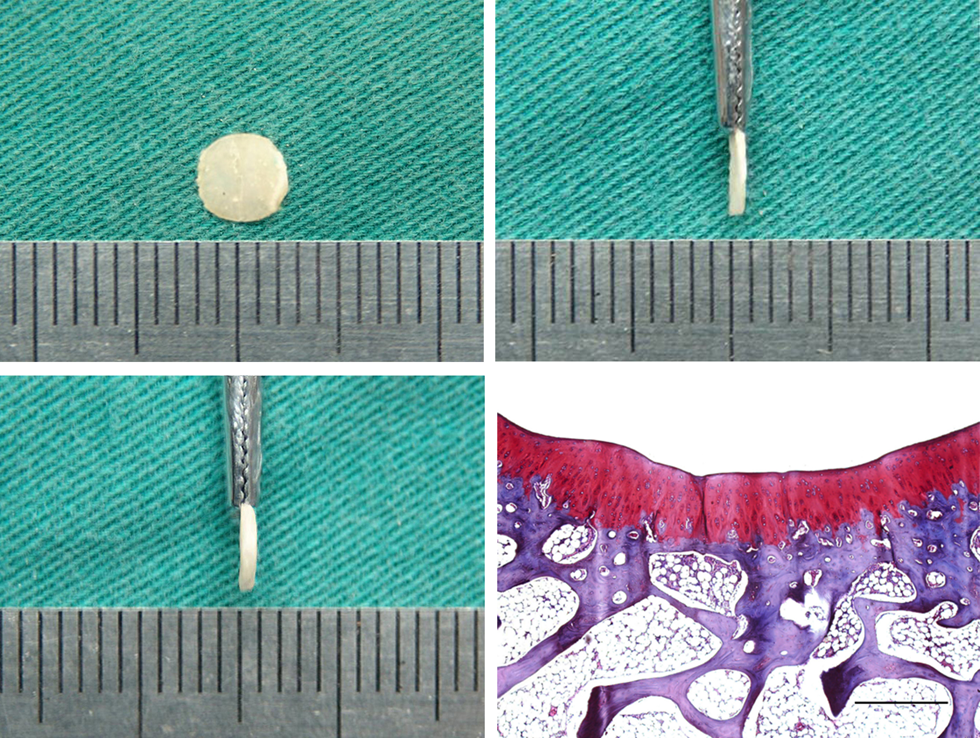

Supplement: Figure S2 — The thickness of the neocartilage. The mean thickness was approximately 0.75 mm. Two methods were used involving (i) the gross appearance and (ii) the histological section (bar = 1 mm). (TIF) [file pone.0054838.s002.tif]

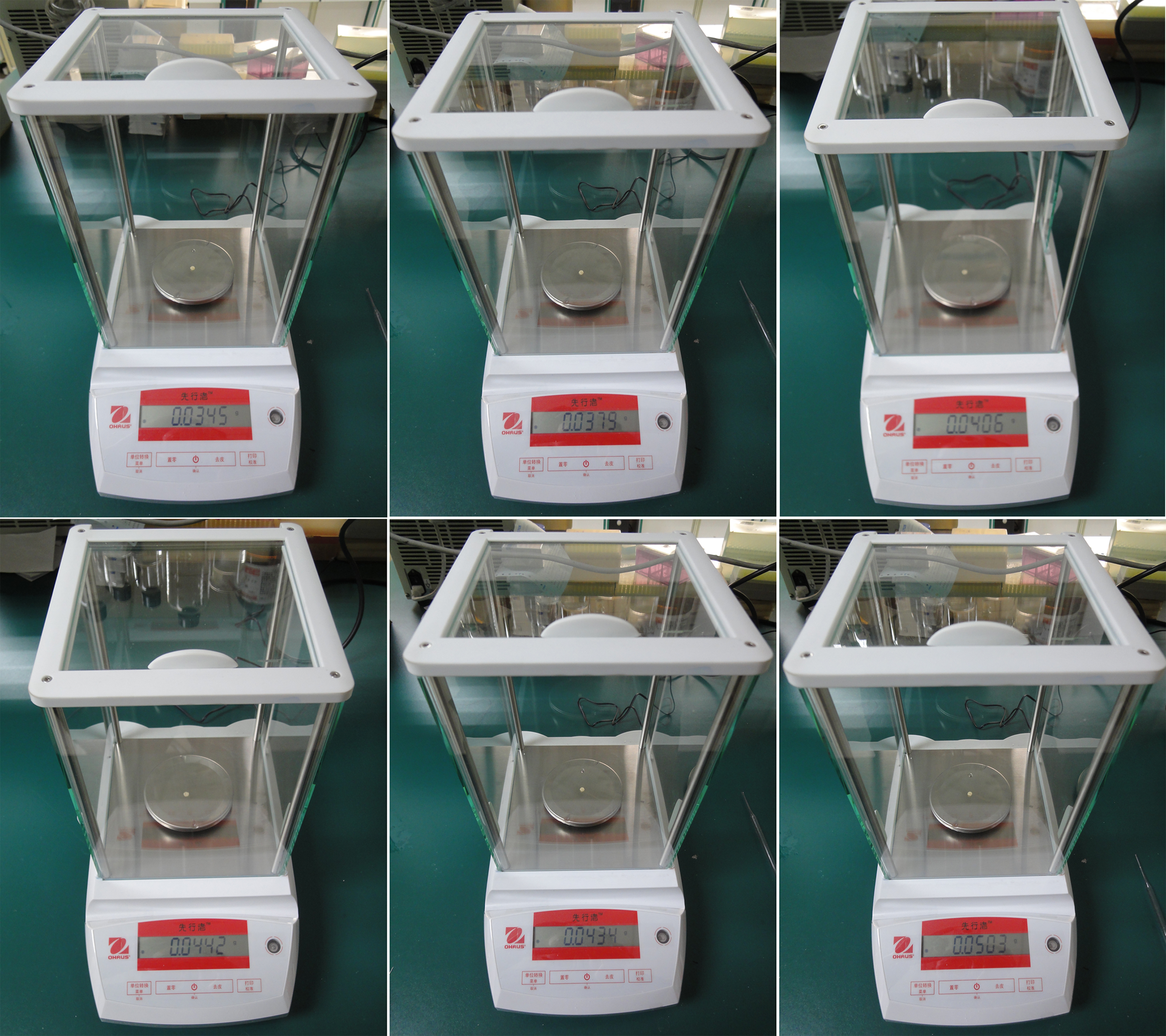

Supplement: Figure S3 — The measurement of the wet weight of some neocartilage. (TIF) [file pone.0054838.s003.tif]

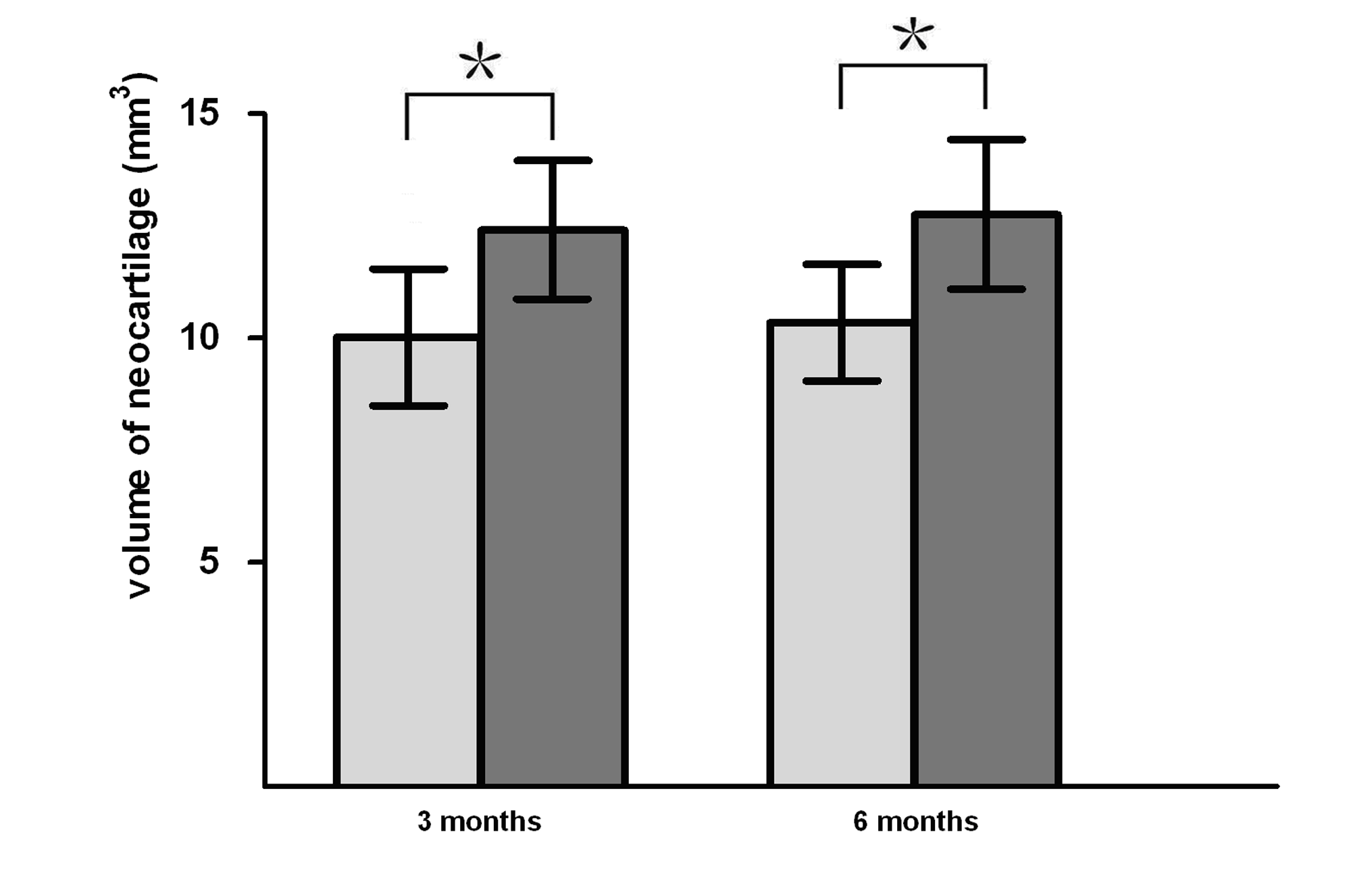

Supplement: Figure S4 — The volume of the neocartilage. The reconstruction utility was used to quantify the volume of the neocartilage. From the reconstruction data, a cylindrical region of interest (ROI) (diameter = 4.5 mm) was selected to analyze the volume of the neocartilage corresponding to the original defect location. (Gray bars represent experimental group, light gray bars represent control group.) (TIF) [file pone.0054838.s004.tif]

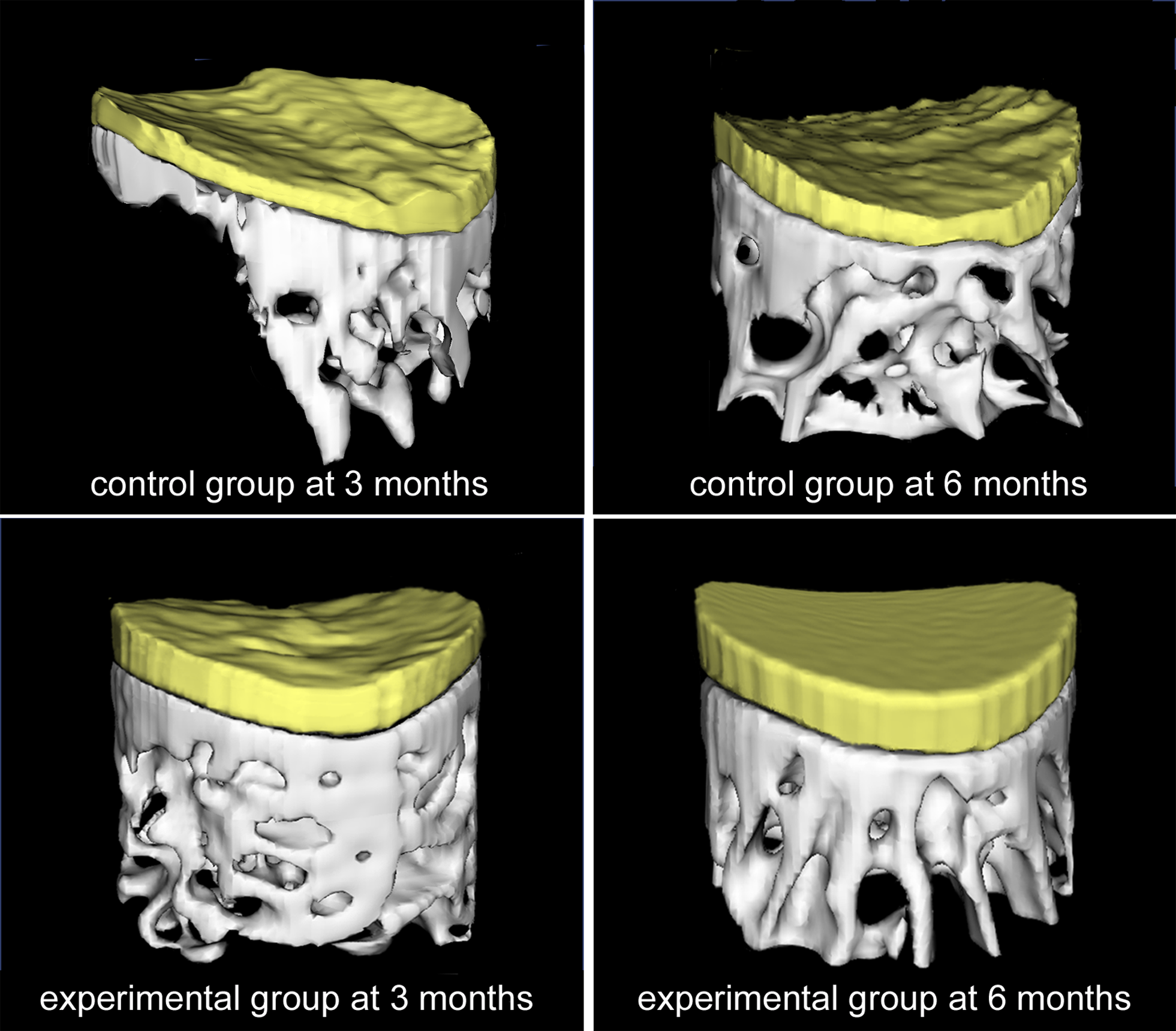

Supplement: Figure S5 — The representative images of the regenerated tissues. (TIF) [file pone.0054838.s005.tif]

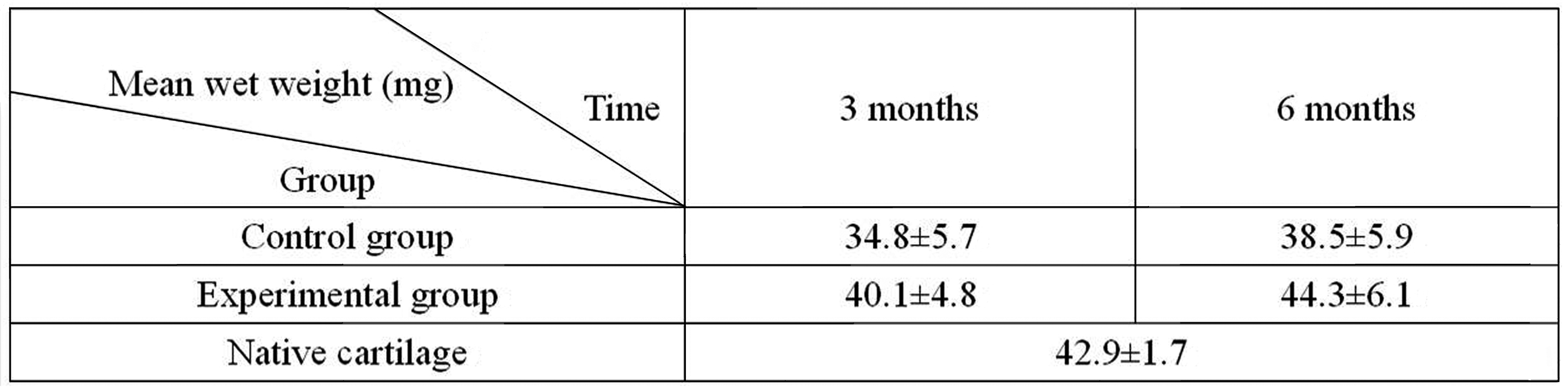

Supplement: Table S1 — The mean wet weight of the neocartilage and native cartilage (diameter = 4.5 mm). (TIF) [file pone.0054838.s006.tif]
